# Blood coagulation factors and platelet response to drug‐induced hepatitis and hepatosis in rats

**DOI:** 10.1002/ame2.12301

**Published:** 2022-12-27

**Authors:** Daria Korolova, Viktoriya Gryshchenko, Tamara Chernyshenko, Oleh Platonov, Olha Hornytska, Volodymyr Chernyshenko, Pavlo Klymenko, Yevdokiia Reshetnik, Tetyana Platonova

**Affiliations:** ^1^ Department of Structure and function of proteins Palladin Institute of Biochemistry of NAS of Ukraine Kyiv Ukraine; ^2^ National University of Life and Environmental Sciences of Ukraine Kyiv Ukraine; ^3^ SI “D.F. Chebotarev Institute of Gerontology of the National Academy of Medical Sciences of Ukraine,” Kyiv Ukraine; ^4^ Taras Shevchenko National University of Kyiv Kyiv Ukraine

**Keywords:** hemostasis, hepatitis, hepatosis, platelets, prothrombin

## Abstract

**Background:**

Knowing the variability of blood coagulation responses to liver damage of different origins can provide a key to curing liver tissues or to mitigating treatment side effects. The aim of the present work was to compare the changes in the main components of hemostasis under experimental drug‐induced hepatosis and hepatitis in rats.

**Methods:**

We modeled diclofenac‐induced hepatitis and tetracycline‐induced hepatosis. Hemostasis response was gauged by measuring fibrinogen, factor X, protein C (PC), and prothrombin in plasma. The decarboxylated form of prothrombin was detected by measuring prothrombin index and ecamulin index. Platelet reactivity was studied using aggregometry.

**Results:**

Both hepatitis and hepatosis decreased the synthesis of fibrinogen, factor X, and prothrombin. However, protein carboxylation was not disrupted in hepatosis but was much impaired in hepatitis. PC decreased in both models as a consequence of its consumption possibly during inflammatory response. Platelet aggregation rate was lower in hepatosis but higher in hepatitis.

**Conclusions:**

Our findings imply the need for a thorough monitoring of the hemostasis system in liver diseases to avoid possible thrombotic complications. Its state indicates the disorder's rate and character.

## Graphical abstract

Head‐to‐head comparison of hemostasis system parameters at drug‐induced hepatitis vs hepatosis in rats was performed. It was assumed that hemostasis system parameters should be monitored thoughtfully during liver diseases to avoid possible thrombotic complications and information about hemostasis system conditions can indicate the rate and character of liver disorders.

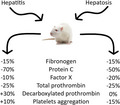

## 1 INTRODUCTION

The liver plays an important role in maintaining blood hemostasis. It participates in carbohydrate, protein, and lipid metabolism, and eliminates various endogenous and exogenous molecules. A multitude of substances, including vitamins (A, D, B12, K, E, and B1), are stored in its cells. The liver is also the site of synthesis of plasma proteins, most coagulation factors, anticoagulant proteins, compounds of fibrinolysis, and stimulators of erythropoesis.^1^

Liver pathologies lead to complex disorders in the blood hemostasis system in which the procoagulant and anticoagulant potentials are imbalanced, causing thrombotic events or hemorrhages.^2^, ^3^

Common agents that damage the liver are medical preparations. Antibiotics, sulfanilamides, hormones, and so on can cause pathological states that occasionally worsen into chronic diseases even more aggravating than the causes for their initial prescription.^4^, ^5^, ^6^

Liver injury leads to the disruption of protein synthesis.^7^, ^8^ Hepatocytes are the main source of clotting factors. The disruption of liver functions leads to changes in the γ‐carboxylation of vitamin K‐dependent factors (mainly factors VII, IX, and X; prothrombin; and proteins С, S, and Z). When decarboxylated, these factors are functionally inactive. They can neither form enzymatic complexes on the surface of the membrane lipid bilayer nor be activated in the physiological clotting cascade.^9^ Their accumulation lowers the procoagulant potential of blood plasma.^10^, ^11^, ^12^

Toxic damage of liver can lead to pathological conditions such as necrosis, cholestasis, fat or toxic hepatitis, cirrhosis, and fibrosis. All these processes imbalance the hemostasis system affecting this very sensitive mechanism on so many levels of regulation that it cannot be easily predicted or healed. Thromboses and hemorrhages are common for liver diseases.^13^, ^14^, ^15^

Hemostasis dysfunctions during liver injuries are broadly studied. However, there is no direct head‐to‐head comparison of how the blood coagulation system (BCS) reacts to modeled liver injuries of different origins. Therefore, the main aim of the present work was to compare the system's response to hepatitis and hepatosis induced in the same rat line. We speculate that the specifics of BCS response to liver injuries of different origins can shed some light on how to eliminate side effects of drugs and cure the liver.

## 2 MATERIALS AND METHODS

### 2.1 Materials

Chromogenic substrates S2765 (Z‐D‐Arg‐Gly‐Arg‐pNA) and S2236 (p‐Glu‐Pro‐Arg‐pNa) were purchased from Diapharma (West Chester Township, OH, USA), and thromboplastin and protein C (PC) activator were obtained from Siemens (Erlangen, Germany). Ecamulin was purified from *Echis multisquamatis* venom according to the method of Solovjov et al.^16^ Factor X activator from *Daboia russellii* venom (RVV [Russell's viper venom]) was purchased from Sigma‐Aldrich (St. Louis, MO, USA).

### 2.2 Animal models

Two different forms of drug‐induced hepatopathologies were modeled: toxic hepatitis and fatty liver disease.

The experiment was performed using male Wistar rats (weight: 200–220 g). For 14 days until the experiment began, the animals were quarantined and clinically examined daily. They were fed a standard diet. Food and drinking water were provided ad libitum. During the quarantine period, we monitored changes in their body weight and feed intake.

The procedures were conducted in accordance with the requirements of the European Convention for the Protection of Vertebrate Animals Used for Experimental and Other Scientific Purposes (Strasbourg, 1986) and the Law of Ukraine on the Protection of Animals from Cruelty (no: 3447 of February 21, 2006).

#### 2.2.1 Model of fatty hepatosis

The rats were divided into two groups: control and experimental (*n* = 10 in each group). For experimental modeling of acute fatty hepatosis according to Gryshchenko et al,^17^ the rats of the experimental group were intragastrically administered 4% aqueous solution of tetracycline hydrochloride once a day for 7 days at 250 mg/kg body weight.

Animals of the control group were intragastrically administered an equivalent volume of double‐distilled water. The experiment lasted 1 week. The biological material was collected on the eighth day under ether anesthesia.

#### 2.2.2 Model of toxic hepatitis

This study also used two groups of rats: control and experimental (*n* = 10 in each group). Toxic hepatitis was induced by intragastric administration of a 4% aqueous solution of sodium diclofenac at 12.5 mg/kg body weight, once a day for 14 days, as described in Serdyukov et al.^18^ The animals of the control group were given an equivalent amount of double‐distilled water intragastrically. The biological material was collected on the 15th day.

### 2.3 Methods

#### 2.3.1 Plasma collection

The blood was collected by puncturing the heart. Sodium citrate (3.8%) was added to the whole blood in the ratio of 1:9 immediately after blood collection. For the aggregometry study, platelet‐rich plasma (PRP) was obtained from whole blood by centrifugation at 160*g* for 30 min at 25°C. PRP was centrifuged at 1300*g* for 15 min at 25°C, and the platelet‐poor plasma (PPP) was collected above the platelet pellet and frozen at −35°C. PPP was thawed at 37°C prior to the measurements.

#### 2.3.2 Biochemical and hematological testing

An automatic hematological analyzer BC 2800 Vet (Mindray, Shenzhen, China) was used to measure blood composition quickly. In particular, it allowed the estimation of the number of white blood cells (WBC); number of lymphocytes, granulocytes, platelets, and red blood cells; percentage of lymphocytes, mid‐sized cells, granulocytes, and platelets; and WBC histogram, hemoglobin concentration, and mean platelet volume.

Biochemical parameters of blood plasma samples were determined using a fully automatic analyzer BioSystem A15 (BioSystems, Barcelona, Spain). We focused on the measuring of alanine aminotransferase (ALT), aspartate aminotransferase (AST), alkaline phosphatase, γ‐glutamyltranspeptidases, total bilirubin, and total protein levels.

The measurements were performed according to the manufacturer's recommendations.

#### 2.3.3 Carracci hematoxylin and eosin staining

Histological samples of the aorta for light‐microscopic examination were prepared as previously described.^17^, ^18^ The samples were stained with hematoxylin and eosin solution according to Carracci and studied using microscopy. Stained histological samples were studied using an Olympus BX51 microscope (Olympus, Tokyo, Japan).

#### 2.3.4 Fibrinogen concentration

Fibrinogen concentration in the blood plasma was determined using the modified spectrophotometric method. The blood plasma (0.2 ml) and phosphate buffered saline (1.7 ml) were mixed in a glass tube. Coagulation was initiated by adding 0.1 ml of thrombin‐like enzyme from the venom of *Aghistrodon halys halys* (1 NIH/ml) to avoid fibrin cross‐linking. The mixture was incubated for 30 min at 37°C. The fibrin clot was removed and dissolved in 5 ml of 1.5% аcetic acid. The concentration of protein was measured using the POP spectrophotometer (Optizen, Daejeon, Korea) at 280 nm (*ε* = 1.5).^19^

#### 2.3.5 Factor X level

Total factor Х level was determined using RVV (reagent that specifically activates factor X, purchased from Sigma‐Aldrich, Saint Louis, MO, USA) and factor Xa‐specific chromogenic substrate S22765 (Z‐D‐Arg‐Gly‐Arg‐pNA).^20^

In a 96‐well plate, 0.02 ml of plasma sample, 0.03 ml of S2765 solution (0.25 mM), and 0.01 ml of RVV solution were admixed in 0.05 M Trisbuffered saline (TBS) (pH 7.4) with 0.13 M NaCl (TBS) and 0.001 М CaCl_2_ to obtain a final volume of 0.25 ml. The synthesis of colorful *p*‐nitroaniline (pNa) was monitored at 405 nm using a ThermoMultiscan (ThermoFisher, Waltham, MA, USA). Results are presented as percentage from control values.

#### 2.3.6 PC level

The total PC level in blood plasma was determined using PC activator and PC‐specific chromogenic substrate S2236 (p‐Glu‐Pro‐Arg‐pNa).^21^

In a 96‐well plate, 0.02 ml of plasma sample, 0.03 ml of S2236 solution (0.25 mM), and 0.03 ml of PC activator solution were admixed in TBS with 0.001 М CaCl_2_ to obtain a final volume of 0.25 ml. The synthesis of colorful pNa was monitored at 405 nm using a ThermoMultiscan (ThermoFisher). Results are presented as percentage from control values.

#### 2.3.7 Prothrombin index

The prothrombin test is based on the application of thromboplastin that acts through the tissue factor pathway of coagulation and activates only the functionally active carboxylated and uncleaved forms of prothrombin.

The results of the prothrombin test were presented as prothrombin index (PI) calculated using the formula PI = An/Ap, where An is the blood plasma clotting time of healthy controls and Ap is the experimental blood plasma clotting time.

#### 2.3.8 Ecamulin index

The test is based on the application of prothrombin activator from the venom of *E. multisquamatis—*ecamulin. Ecamulin activates prothrombin, descarboxy‐prothrombin, and prethrombin 1, thus permitting the determination of total prothrombin level.^22^

The results of the ecamulin test were presented as ecamulin index (EI) calculated using the formula EI = An/Ap, where An is the blood plasma clotting time of healthy controls and Ap is the experimental blood plasma clotting time.

#### 2.3.9 Platelet aggregation

Platelet aggregation was measured based on changes in the turbidity of PRP.^23^ In a typical experiment, 250 μl of PRP was incubated with 25 μl of 0.025 M CaCl_2_ and 25 μl of 12.5 μM adenosine diphosphate at 37°C. Aggregation was monitored for 10 min using the aggregometer Solar 2110 (Solar, Minsk, Belarus).

#### 2.3.10 Statistics

Statistical data analysis was performed using the Kruskal–Wallis test (https://www.socscistatistics.com/tests/kruskal/default.aspx). All blood coagulation assays were replicated thrice. The results are presented as boxplot diagrams with median, maximal and minimum values, and interquartile range. The results were considered significant at *p* < 0.05.

The Wilcoxon‐Mann‐Whitney test was used to estimate and compare the differences between two independent groups. This is widely used for comparisons of small groups by the level of any qualitatively measured attribute and allows to identify differences in the value of parameter.^24^,^1^

## 3 RESULTS

### 3.1 Modeling of hepatosis and hepatitis

Administration of tetracycline for 7 days led to the complexity of characteristic symptoms of the acute stage of fatty hepatosis.^17^ Depression, loss of appetite, thirst, weight loss by 10–15 g, dimming of the wool, and so on, were observed starting from the third day of the experiment. Analysis of blood cells demonstrated an increase in leukocytes by 27% (6.1 ± 0.5 × 10^9^/L vs. 4.8 ± 0.2 × 10^9^/L in the control group), a decrease in red blood cells by 27% (4.8 ± 0.3 × 10^12^/L vs. 6.6 ± 0.5 × 10^12^/L in the control group), and a decrease in hemoglobin level by 23% (141.0 ± 3.6 g/L vs. 182.7 ± 12.1 g/L in the control group). Hematocrit decreased from 35.2 ± 2.3% to 27.2 ± 1.7%, whereas plateletcrit increased (0.322 ± 0.016% vs. 0.249 ± 0.018% in controls).

We also observed an increase in the segmentoid neutrophils (36.0 ± 0.4% vs. 33.5 ± 0.8% in the control group) and a decrease in rod‐shaped neutrophils (5.5 ± 0.5% vs. 8.0 ± 0.2% in the control group). The 2.3‐fold increase in eosinophils (3.5 ± 0.5% vs. 1.5 ± 0.2%) in the hepatosis model can be explained by their well‐known antitoxic function.

ALT and AST activities increased from 34.10 ± 3.32 to 102.30 ± 2.39 IU and from 70.24 ± 3.11 to 127.81 ± 4.05 IU, respectively. Activity of alkaline phosphatase increased by 60% (400.11 ± 9.81 vs. 251.01 ± 10.01 IU in controls) and γ‐glutamyltranspeptidases by 73% (21.33 ± 1.11 vs. 12.33 ± 0.69 IU in the control group).

Total bilirubrin level increased 15 times and reached 45.5 ± 3.0 μmol/L (3.0 ± 0.2 μmol/L in the control group). The conjugated fraction contributed the most to this increase (27.34 ± 0.83 vs. 1.46 ± 0.24 μmol/L).

These findings confirmed the destructive changes in hepatocytes, decrease in protein synthesis, disruption of pigment metabolism, and signs of cholestasis during the development of experimental drug‐induced hepatosis modeling.^7^, ^17^

The development of the experimental drug‐induced hepatitis^18^, ^25^ model provoked changes in hematological and protein profiles. Pronounced clinical symptoms of the disease began to appear on the sixth day of daily administration of diclofenac. In particular, we observed general depression and immobility, loss of appetite, decreased average body weight by 15–20 g, decrease in skin elasticity, increase in the sensitivity of the abdomen walls, and so on.

Hematological analysis detected leucocytosis by the increasing number of leucocytes from 5.7 ± 0.2 × 10^9^/L in the control group to 17.10 ± 0.8 × 10^9^/L with consequent shift of the neutrophil nucleus to the right side and a 2.6‐fold decrease in the number of monocytes (3.6 ± 0.1% vs. 9.3 ± 0.3% in the control group). We also detected a decrease in the number of red blood cells (6.9 ± 0.2 × 10^12^/L vs. 3.9 ± 0.1 × 10^12^/L in controls), yet hematocrit and hemoglobin levels were unchanged. All this suggested an inflammation process and anemia.^26^

The total protein concentration decreased from 41.3 ± 1.6 to 36.8 ± 0.7 g/L. The ALT and AST activities increased from 91.5 ± 6.6 to 17.3 ± 7.2 IU and from 89.2 ± 4.8 to 129.4 ± 5.8 IU, respectively. The activity of alkaline phosphatase increased by 30% (456.2 ± 16.1 vs. 350.3 ± 15.2 IU in the control group), and that of γ‐glutamyltranspeptidases increased 2.1‐fold (24.1 ± 0.6 vs. 11.3 ± 0.4 IU in the control group).

These changes indicate pathological changes in liver function, including the disruption of pigment metabolism and cholestasis.^27^

**FIGURE 1:**
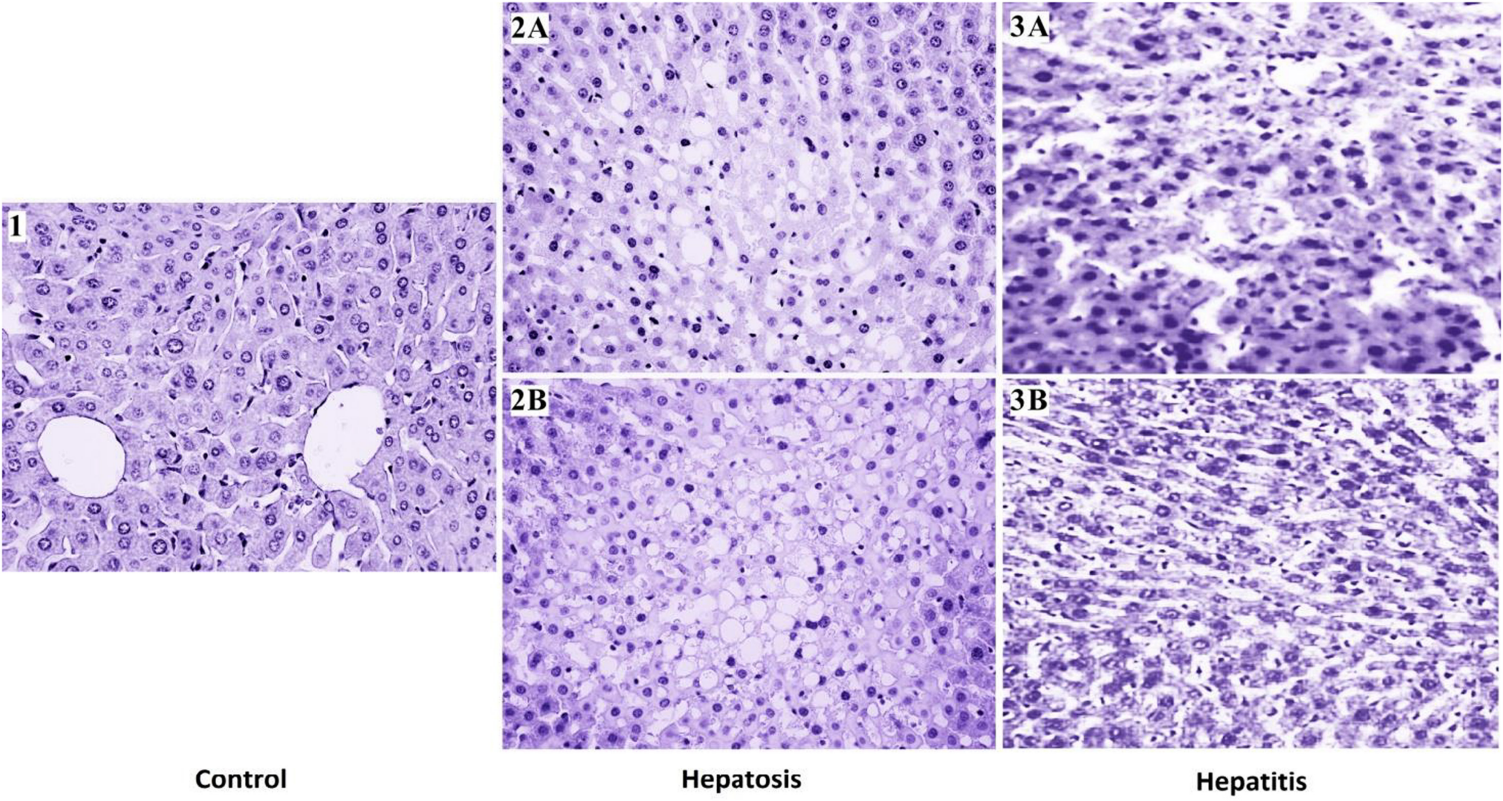
Typical histological analysis of liver of rats with drug‐induced hepatosis or hepatitis in comparison to liver of control rat. Stained with Carracci hematoxylin and eosin, 200×. Control. A liver of a rat from one of the control groups. Note the central veins of liver lobules with radial rows of hepatocytes. The liver has a typical healthy structure. Hepatosis. 2A,B. Rat liver under experimentally induced adipose hepatosis. Accumulation of fat in the pericellular matrix, the substitution of capillars by fat infiltrations. Liver parenchyma partly substituted with fat infiltrations. Condensation of chromatin in cell nuclei. Hepatitis. 3A,B. The disruption of liver cytoarchitecture, loss of structure of liver tissue, heterochromatization of cell nuclei. Expanded capillary space.

Morphological changes in liver during hepatosis or hepatitis are shown in Figure 1.

Tetracycline has a direct cytotoxic effect on the liver. It first manifests as a fatty dystrophy with the accumulation of triglycerides in hepatocytes and the disruption of the balance between the production and catabolism of lipids. At high doses, it causes a decrease in the activity of mitochondrial beta‐oxidation of fatty acids and an increase in the synthesis of endogenous fatty acids and leads to insufficient incorporation or export of triglycerides into low‐density lipoproteins.

Macroscopic changes in the liver with tetracycline‐induced hepatosis are described in Gryshchenko et al.^17^ Microscopic examination of the organ showed decomposing hepatocytes, diffuse placement of lipid droplets of varying size in the cells' cytosol, focal histiolymphocytic infiltration, and dilation and overflow of blood vessels. In addition, liver functions were disrupted (protein synthesis, energy, regulatory, pigmentary, and biliary excretion).^17^

Toxic damage to the liver by sodium diclofenac affects the mitochondrial and endoplasmic membranes of hepatocytes. It intensifies lipid peroxidation, increasing the total and free cholesterol and also the phospholipids. Simultaneously, diclofenac disrupts detoxification and protein synthesis in the liver, damaging cellular and subcellular membranes.

In this model, rats exhibited characteristic macroscopic changes in the liver^18^, ^27^: flabby texture and signs of dystrophy. Microscopic examination of organ sections in sick animals revealed dilated blood vessels overflowing with blood, individual cells in the state of fat degeneration (nuclei were displaced to the periphery of the cell, cytoplasm was transparent, hepatocytes were ring shaped), severe edema in the Disse space, discomplexation of liver beams, and lymphocytic infiltration of connective tissue, which confirms the development of an inflammatory reaction. Discomplexation encompassed certain areas of the liver lobes; hepatocytes were randomly arranged and formed cellular structures. Simultaneously, the hepatocytes' metabolic and functional activities changed, as evidenced by hypoproteinemia, hypoalbuminemia, hypoglycemia, hypolipidemia, hypocholesterolemia, and high thymol test values.

### 3.2 BCS parameters

To evaluate the condition of the BCS we measured the main coagulation factors (fibrinogen, prothrombin, and factor X) and the crucial anticoagulant factor PC.

**FIGURE 2:**
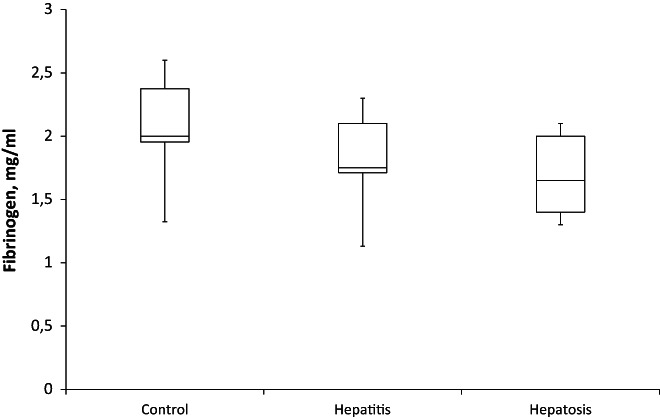
Concentration of fibrinogen in blood plasma of rats with experimental drug‐induced hepatosis (*n* = 10) and hepatitis (*n* = 10). Here and later only one control is presented, as the control parameters were similar in both. The *p*‐value is 0.16525. The result is not significant at *p* < 0.05.

We found a tendency to reduce 15%–20% in fibrinogen level in blood plasma of both experimental groups compared to controls (Figure 2). This was in spite of inflammatory processes accompanying liver injuries and can be associated with the suppression of protein synthesis.

**FIGURE 3:**
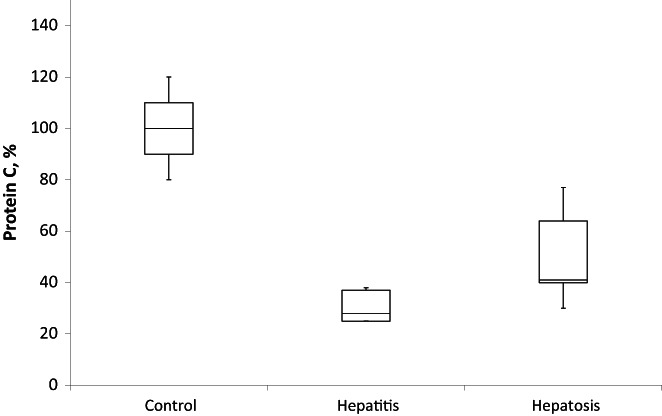
Protein C in blood plasma of rats with experimental drug‐induced hepatosis (*n* = 10) and hepatitis (*n* = 10). The *p*‐value is < 0.00001. The result is significant at *p* < 0.05.

**FIGURE 4:**
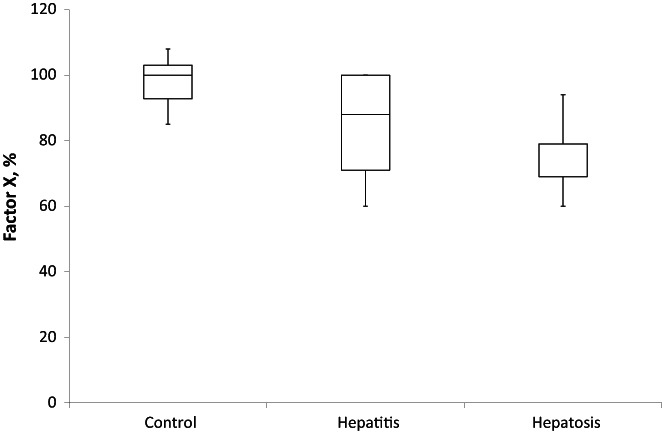
Clotting factor X in blood plasma of rats with experimental drug‐induced hepatosis (*n* = 10) and hepatitis (*n* = 10). The *p*‐value is 0.00593. The result is significant at *p* < 0.05.

Decrease in the plasma content of factor X and PC, which are also produced by the liver, confirmed this suggestion. Moreover, total PC decreased from 100 ± 10% in controls to 30 ± 6% and 51 ± 15% in the hepatosis and hepatitis groups, respectively (Figure 3). Decrease in the level of total factor X was less obvious. However, it was reduced up to 40% in both models and reached 84 16% in hepatitis and 77 ± 12% in hepatosis groups versus 100 ± 10% in controls (Figure 4).

The liver is also responsible for carboxylation of PC, fibrinogen, prothrombin, and factor X. In the case of prothrombin we were able to evaluate its total content as well as the content of carboxylated forms using a combination of two tests: EI and PI.

**FIGURE 5:**
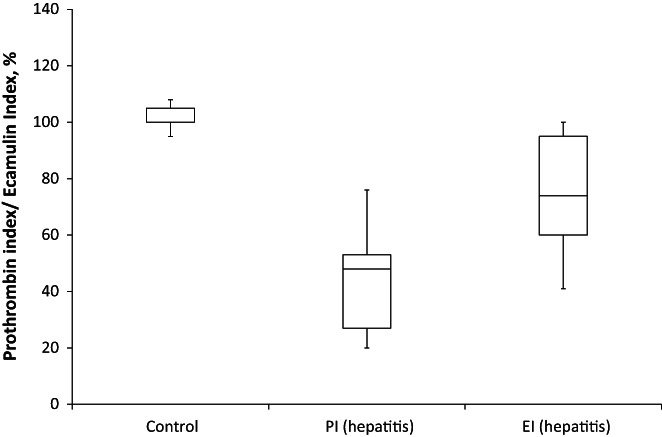
Prothrombin index and ecamulin index in blood plasma of rats with experimental drug‐induced hepatitis (*n* = 10). The *p*‐value is 0.00001. The result is significant at *p* < 0.05.

**FIGURE 6:**
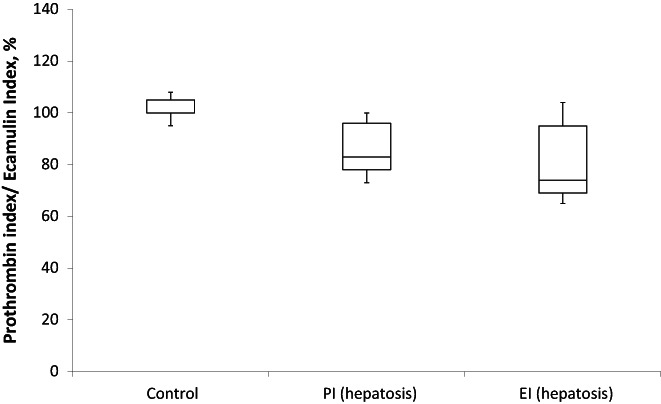
Prothrombin index and ecamulin index in blood plasma of rats with experimental drug‐induced hepatosis (*n* = 10). The *p*‐value is 0.0014. The result is significant at *p* < 0.05.

By applying EI we demonstrated a decrease in the total content of prothrombin—to 75 ± 20% in hepatitis and 80 ± 14% in hepatosis, similar to factor X and PC (20%–30%) (Figures 5 and 6). The use of PI also confirmed that prothrombin was not only less synthesized but also much less carboxylated in the case of hepatitis compared to controls (Figure 4). Simultaneously, hepatosis affected the content of prothrombin but not its carboxylation (Figure 5).

**FIGURE 7:**
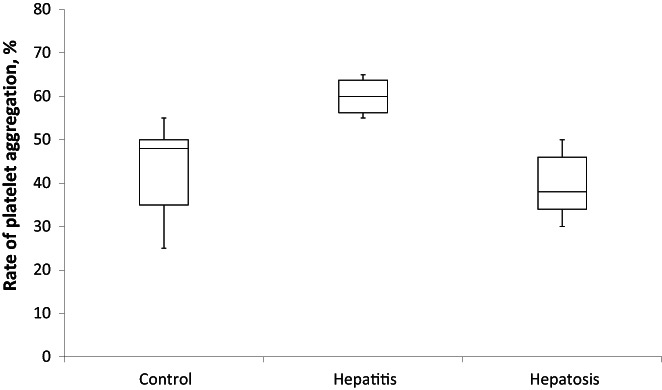
Rate of platelet aggregation in platelet‐rich blood plasma of rats with experimental drug‐induced hepatitis (*n* = 10) and hepatosis (*n* = 10). The *p*‐value is 0.00113. The result is significant at *p* < 0.05.

Pathological changes in protein synthesis and carboxylation during drug‐induced hepatitis were also accompanied by a statistically significant increase in the rate of platelet aggregation (up to 60 ± 4.5% compared to 43 ± 11% in control). In contrast, hepatosis provoked the decrease in platelet aggregation rate to 39 ± 6.5% (Figure 7).

## 4 DISCUSSION

Liver diseases are dangerous and spread widely, leading to acute liver failures and thus impairing the quality of human life.^28^, ^29^, ^30^ Drug hepatotoxicity can also be a reason for acute liver failure. The symptoms can manifest immediately or several months into therapy. Accurate diagnostics can be a basis for the selection of appropriate hepatoprotective therapy.^4^, ^31^, ^32^

The pathophysiological state of the liver leads to disorders in many systems of the human body, including the very sensitive and important system of hemostasis. In our study we performed complex hemostasis analysis and confirmed significant changes in hemostasis in rat models of two drug‐induced liver pathologies: hepatosis and hepatitis. Some of these changes (e.g., the decrease in PC or hyperaggregation of platelets) are alarming signs of hypercoagulation and the risk of thrombosis or bleeding.^33^, ^34^, ^35^

These findings result in two main assumptions: (1) hemostasis system parameters should be monitored thoroughly during liver complications to avoid possible thrombotic complications, and (2) information on BCS conditions indicates the rate and characteristics of liver disorders.

According to our data, liver diseases result in a decrease in main blood coagulation factors (factor X, prothrombin, and fibrinogen). The most obvious decrease was observed in the case of the main anticoagulant proenzyme PC. Its level decreased also because of the consumption during intravascular thrombin generation and inflammation.^36^, ^37^ We observed that both hepatitis and hepatosis decreased the synthesis of fibrinogen, factor X, and prothrombin. However, the results of the prothrombin and ecamulin tests suggest that carboxylation of proteins was not disrupted in hepatosis, so the vitamin K‐dependent proteins in this pathology are functionally active. In the case of hepatitis, the functional properties of proteins were dramatically disordered due to the production of their decarboxylated forms that cannot be involved in the coagulation cascade in physiological pathways.

We also detected a tendency toward a lower platelet aggregation rate in the case of hepatosis, which can be assumed as a predisposition to bleeding. However, this fact must be evaluated in complex with other parameters of blood coagulation.

On the contrary, the increase in platelet aggregation rate during hepatitis confirms the imbalance in hemostasis, which must be studied more precisely.

We conclude that the determination of the rate of total synthesis and carboxylation of clotting factors can provide vital information on liver condition and the risk of bleeding/thrombosis. Therefore, we recommend detecting the decarboxylated forms of prothrombin during liver diseases using all validated methods available.^9^, ^38^, ^39^ PC level should also be estimated during liver diseases as the most sensitive factor that can indicate the severity of liver disease and also the risk of intravascular coagulation. Finally, platelet aggregation should be considered as an additional functional test for analyzing the risk of intravascular coagulation during liver diseases.^40^, ^41^

Newly established differences between BCS responses to hepatosis and hepatitis indicate the necessity to study the pathological mechanisms of these diseases to find a method to avoid complications.

## AUTHOR CONTRIBUTIONS

**Daria Korolova**: manuscript preparation, methodology, measurements, and aggregometry; **Viktoriya Gryshchenko**: methodology, animal model development, and ideology of study; **Tamara Chernyshenko**: laboratory tests; **Oleh Platonov**: visualization and data analysis; **Olha Hornytska**: manuscript preparation; **Volodymyr Chernyshenko**: concept of the article and review and editing; **Pavlo Klymenko**: histology; **Yevdokiia Reshetnik**: animal model development; **Tetyana Platonova**: supervision and ideology of the study.

## FUNDING INFORMATION

National Academy of Sciences of Ukraine research grant number 0119U002512.

## CONFLICT OF INTEREST

The authors declare no competing interests.

## ETHICS APPROVAL

All animal studies were carried out in accordance with the standards of the "European Convention for the Protection of Vertebrate Animals Used for Experimental and Other Scientific Purposes" (Strasbourg, 1986), "Convention on Bioethics of the Council of Europe" (1997), "General Ethical Principles for Experiments on Animals", adopted by the First National Congress of Ukraine on Bioethics (2001), other international agreements and national legislation in this area. Blood samples were taken from animals using inhalation chloroform anesthesia. This study was carried out with the approval of the Animal Care and Use Committee of the Palladin Institute of Biochemistry, National Academy of Sciences of Ukraine (Protocol N5 from 07/05‐2018).

## Footnotes

1 If it will not be a problem, could You please delete this paragraph? Thank You in advance.
